# Exploring the opinions and potential impact of unflavoured e-liquid on smoking cessation among people who smoke and smoking relapse among people who previously smoked and now use e-cigarettes: findings from a UK-based mixed methods study

**DOI:** 10.1186/s12954-024-01003-z

**Published:** 2024-05-03

**Authors:** Jasmine N. Khouja, Maddy L. Dyer, Michelle A. Havill, Martin J. Dockrell, Marcus R. Munafò, Angela S. Attwood

**Affiliations:** 1 School of Psychological Science, University of Bristol, 12a Priory Road, Bristol, BS8 1TU UK; 2 Medical Research Council Integrative Epidemiology Unit, University of Bristol, Bristol, BS8 2BN UK; 3 Department of Health and Social Care, Office of Health Improvement and Disparities, London, SW1H 0EU UK; 4 NIHR Biomedical Research Centre at the University Hospitals Bristol NHS Foundation Trust and School of Psychological Science, University of Bristol, 12a Priory Road, Bristol, BS8 1TU UK

**Keywords:** e-cigarette, Vaping, Smoking, e-liquid flavour, Flavour restriction, Flavour ban, Gateway hypothesis

## Abstract

**Background:**

Although electronic cigarettes (e-cigarettes) appear to be effective in helping people who smoke to stop smoking, concerns about use of e-cigarettes among young people have led to restrictions on non-tobacco flavoured e-liquids in some countries and some US states. These restrictions could reduce the appeal of these products to non-smoking youth but could have negative consequences for people who smoke or use e-cigarettes.

**Methods:**

In this mixed methods study, we recruited UK adults who smoked or used to smoke and subsequently vaped to explore their opinions of unflavoured e-liquids and their beliefs about how they would be impacted by hypothetical e-liquid flavour restrictions. Participants trialled an unflavoured e-liquid instead of their usual nicotine product for four hours and completed a survey and an online interview.

**Results:**

Using Interpretive Phenomenological Analysis and graphically presented data, we found differences in participants’ opinions of unflavoured e-liquid. If only unflavoured, tobacco flavoured, and menthol flavoured e-liquids remained on the UK market, some people who smoke or vape may be unaffected, but some may relapse to smoking or continue smoking. Despite most wanting to prevent young people from initiating vaping, participants had varying opinions on whether flavour restrictions would be an effective method.

**Conclusions:**

The findings highlight that people who smoke and vape could be impacted by flavour restrictions in a range of ways, some of which could have a potential adverse impact on harm reduction efforts in the UK (e.g., by making smoking more appealing than vaping).

**Supplementary Information:**

The online version contains supplementary material available at 10.1186/s12954-024-01003-z.

## Background

Electronic cigarettes (also known as e-cigarettes or vapes) are battery-operated devices that heat a liquid (also known as e-liquid) to create an aerosol, which can be inhaled. Using e-cigarettes is sometimes referred to as ‘vaping’ [33]. With approximately 4.7 million people who vape in Great Britain, many people use e-cigarettes to cut down or stop smoking [4]. A living systematic review of e-cigarette use for smoking cessation suggests that e-cigarettes are an effective smoking cessation aid [31]. The efficacy of e-cigarettes as smoking cessation tools may be partly dependent on the array of flavours available to aid or maintain smoking reduction or cessation [23], but some countries and US states have policies restricting e-liquid flavour availability. At a federal level, the USA only permits tobacco and menthol flavours in certain products, but in some US states this flavour ban applies to all e-cigarette products, and Finland only permits tobacco flavours. In January 2024, the UK government announced they will be introducing new powers to restrict flavours in e-cigarettes. Given that e-cigarettes have the potential to reduce harm at a population-level, it is important to understand the impact that restrictions may have on people who smoke and people who have quit smoking and switched to vaping in the UK.

E-liquid restrictions have been implemented in some countries and US states due to the belief that flavoured e-liquids appeal to non-smoking youth [47]. It has been suggested that e-cigarettes may attract youth who have never smoked, and that using e-cigarettes could lead to smoking initiation, commonly known as the “gateway effect” [14]. Research has found a strong positive association between e-cigarette use and later smoking among individuals who have not smoked prior to using e-cigarettes [5]. However, evidence from time-series analyses in England have not supported this theory [6] and the association could be due to the two behaviours sharing a common liability, for example a propensity to risk-taking [28]. Nevertheless, these concerns have led to e-liquid flavour restrictions in some countries and US states, and similar restrictions have been announced in the UK. This is despite little being known about the potential negative unintended public health consequences of such restrictions for UK adults who smoke or previously smoked and now vape.

Restrictions on the sale of flavoured e-liquids could result in people who transitioned from smoking to vaping returning to smoking (i.e., relapsing). Just under one in five people who use e-cigarettes surveyed in Great Britain stated that they would smoke more or revert to smoking if flavours were no longer available [3]. People who smoke may also be less interested in using e-cigarettes to stop smoking without the availability of a range of flavours.

If the number of adults who return to smoking or decide not to stop smoking using an e-cigarette outweighs the number of young people who are protected from vaping (and potentially subsequent smoking), then restrictions of e-liquid flavours could result in a net increase in the number of people who smoke in the population [24]. Current evidence suggests that cigarettes pose a much greater health risk than e-cigarettes [34], so the number of young people protected may need to substantially outweigh the number of people who return to or continue smoking cigarettes, to result in a net decrease in population harm. Alternatively, if people who smoke or vape positively perceive e-liquids which would remain on the market in the event of a flavour restriction (e.g., unflavoured e-liquid), then the overall impact of e-liquid flavour restrictions on people who smoke or used to smoke and now vape could be negligible. If they believe that flavour restrictions would have little impact on their behaviour (i.e., they would be just as likely to attempt to stop smoking and would be no more likely to return to smoking) then flavour restrictions could result in fewer people smoking in the population. Although unflavoured e-liquids (i.e., e-liquids containing propylene glycol, vegetable glycerin and nicotine without flavourings) would be available if these hypothetical restrictions were implemented, only 1.4% of adults who vaped in the UK in 2023 reported using unflavoured products [4], so some people who vape or smoke in the UK could be undecided about unflavoured e-liquid.

Understanding the opinions of people who smoke or previously smoked and now vape about unflavoured e-liquids could inform policies. It is also important to understand how people who smoke or previously smoked and now vape believe their future e-cigarette use, smoking behaviours, and behavioural intentions may be impacted by e-liquid flavour restrictions (e.g., banning all e-liquid flavourings except menthol and tobacco). There have been few qualitative explorations of the impact of e-liquid flavour bans or restrictions. One study focused on young US adults who smoked and vaped, finding that banning or restricting flavours (aside from tobacco, menthol or unflavoured e-liquids) could discourage them [15]. Another study among young adults in China who vaped daily for at least three months found they had used a range of adaptative strategies in the 1–3 months since [52]. These strategies included sourcing illegal products and using custom-made cartridge covers which added flavours to add flavours to legal products. After a restriction on flavoured cartridge-based vaping products in the US, young adults reported stockpiling, buying illegal products online, switching to legal flavours, and reducing use, but stated they might stop vaping, switch to cigarettes, or stockpile if flavours were comprehensively restricted in *all* *vaping products* [41]. These results cannot be generalised to adults who smoke or vape in other countries with different regulations, available products, and societal contexts. Among i) adults who currently smoke and ii) adults who currently vape (who have stopped smoking within the last 12 months) in the UK, we aimed to explore: 1) their opinions of unflavoured e-liquid after a brief trial (4 h) of an unflavoured e-liquid, and 2) how participants believe a hypothetical e-liquid flavour restriction (i.e., banning non-tobacco and non-menthol flavoured e-liquids) may impact their future smoking behaviour, vaping, and future intentions to vape unflavoured e-liquids.

## Methods

### Design

This exploratory observational study using mixed methods followed the methods outlined in our online pre-registered study protocol (https://osf.io/snmp9), except where specified. Ethics approval was obtained from the University of Bristol School of Psychological Science Human Research Ethics Committee, a subcommittee of the Faculty of Life Sciences Ethics Committee (reference: 010421116008).

### Participants

We recruited 24 healthy UK residents between April 2021 and July 2021—12 adults who smoked daily and 12 adults who vaped daily (who stopped smoking within the 12 months prior to the study session) as daily vaping is strongly associated with smoking cessation and daily smoking is associated with using a quit aid in a smoking cessation attempt [25, 48]. We recruited four people who smoked 20 or more cigarettes per day (CPD) and two people who vaped daily but used to smoke 20 or more CPD (two fewer than stated in our preregistered protocol due to difficulties in recruitment). A previous qualitative study exploring perceptions of e-liquid flavours among young adults who both smoked and vaped in the US included 25 interviews [15], therefore, we anticipated that 24 interviews would be sufficient to achieve saturation of themes [50]. Participants were recruited through existing email lists, social media (Facebook and Twitter adverts), word of mouth, and via the University of Bristol Tobacco and Alcohol Research Group (TARG) newsletter and website.

Participants were 18 years of age or older, fluent in English, and they self-identified as either a person who smoked daily or a person who vaped daily. Daily smoking was defined as currently smoking five or more times per day for three months or more. Participants who smoked were not currently attempting to stop smoking (i.e., not currently using nicotine replacement products or in active smoking cessation treatment) and were not currently vaping. Daily vaping was defined as currently using a nicotine-containing e-cigarette five or more times per day for three months or more. Participants who vaped daily and previously smoked had recently stopped smoking. This was defined as having previously met the criterion of currently smoking in the 12 months before the study and having replaced smoking with use of an e-cigarette for at least one month before the study. From here on, we refer to these participants as “participants who vaped”. Participants who vaped were required to currently only be using non-tobacco and non-menthol flavoured e-liquids (e.g., fruit flavoured e-liquids). Full eligibility criteria are listed in Additional file 1 (Sect. 1.1). All eligibility criteria were assessed via self-report, and nicotine use and pregnancy criteria were verified using self-administered urine tests. Presence of cotinine in urine, a highly specific biomarker for nicotine [7], was used to confirm current nicotine use.

### Measures and materials

#### E-cigarette and e-liquid

Participants received an Arc 5 starter kit purchased from Totally Wicked (https://www.totallywicked-eliquid.co.uk/arc-5). Each kit contained one e-cigarette with a 2200 mAh internal battery, a CS Air Slim tank, and an atomizer head, a USB charging cable, and a user manual. A tank-style device was selected as it was the most popular device type among adults who regularly vape in Great Britain at the time of the study [3]. Each participant received one 10 ml bottle of unflavoured Red Label e-liquid (50:50 PG/VG) (https://www.totallywicked-eliquid.co.uk/unflavoured-red-label. The e-liquid contained one of two nicotine concentrations (10 mg/ml or 18 mg/ml that best reflected a participant’s typical nicotine use (based on CPD or usual e-liquid nicotine concentration; Additional file 1, Sect. 1.2).

#### Measures

Age, gender, ethnicity, and where applicable, frequency and duration of current and/or past smoking, frequency and duration of current and/or past e-cigarette use, and time since smoking cessation were recorded in a Qualtrics survey [40] (Additional file 2: Tables S1 and S2). Descriptive quantitative data on participant characteristics and perceptions of unflavoured e-liquids prior to exposure were collected. Items measured participants’ willingness and intentions to use unflavoured e-liquids if flavoured e-liquids (i.e., non-tobacco, non-menthol flavoured) were restricted. Participants who smoked were asked if they would be willing to attempt to stop smoking using an e-cigarette, and with unflavoured e-liquid. Motivation to quit smoking was measured using the readiness to quit ladder [1]. The readiness to quit ladder ranges from 1 (“I have decided not to quit smoking for my lifetime. I have no interest in quitting”) to 10 (“I have quit smoking”). Participants who vaped were asked to report how many times per day they used their e-cigarette and were advised to assume that one ‘time’ lasts for around 10 min or consists of around 15 puffs [21]. Participants who vaped were asked: (a) if they believed they would have quit smoking using an e-cigarette if they had used an unflavoured e-liquid, (b) if they thought they would switch to using an unflavoured e-liquid if flavours were restricted, and (c) if they thought they would relapse to smoking instead of switching from a flavoured to an unflavoured e-liquid if e-liquid flavours were restricted. They were then asked what they would do if flavoured e-liquids were removed from the market (e.g., no sweet or fruit flavours), and only tobacco, menthol/mint and unflavoured e-liquids were available. They could select multiple answers from the options provided, and/or insert another answer. These questions and response options are described in full in Additional file 2: Tables S1 and S2.

#### Interview

The semi-structured interview included open-ended questions that were intended to encourage the participants to discuss: (1) their experience and opinions of using unflavoured e-liquid, and (2) how they perceived a restriction on e-liquid flavours may impact their future smoking behaviour, vaping, and future intentions to vape unflavoured e-liquids. Specifically, participants were asked to consider a proposed scenario in which “flavoured e-liquids were removed from the market, and only unflavoured, tobacco or menthol/mint flavours were available”. We additionally asked about their previous experiences with smoking and vaping, and their general thoughts on the hypothetical proposed restrictions. The full interview schedule (including topic guide) is included in Additional file 2: Tables S3 [for people who smoked] and S4 [for people who vaped]). The topic guide was developed based on existing literature [12, 54] and known evidence gaps (detailed in the introduction).

### Procedures

Potential participants self-reported their eligibility to participate during a telephone screening and completed an online written consent form via Qualtrics. Participants provided a postal address. This first session lasted ~ 20 min. Following the telephone screening, the researcher posted an e-cigarette starter kit, objective screening tests (for cotinine and pregnancy, if female), instructions, a cleaning wipe (for the device), and a cover letter to the participant via Royal Mail. The e-cigarette voltage was set to 12W, but participants were instructed to modify this if desired.

Approximately one week later, on the morning of their test session day, the participant completed the objective screening measures (a cotinine test and if female, a pregnancy test). These screening sessions were via video call [55] and were scheduled between 8 am and 1 pm on the same day of the urine test(s). In the final screening session, the participant showed their cotinine (and if applicable, pregnancy) test results to the researcher on camera. Eligible participants were sent a link to an online Qualtrics survey via email to progress to Session 1 of the study. The survey assessed participant characteristics and participants’ perceptions of unflavoured e-liquids and e-liquid flavour restrictions. Following survey completion, the researcher instructed the participant to set up their e-cigarette, fill it with the e-liquid provided, and use this device instead of smoking or using their usual vaping products until Session 2 (~ 4 h later). Final screening and Session 1 were completed during one video call lasting ~ 20 min.

In Session 2, participants reported their puff count (recorded by the e-cigarette), provided verbal consent to begin the audio recording of the interview, and completed a semi-structured interview via a video call [55]. After the interview, participants were emailed a debrief sheet and a voucher for participation (worth £20). Interviews usually lasted 15–25 min and were transcribed verbatim.

### Analyses and interpretation

We report all quantitative data for participants who smoked and participants who vaped separately due to differences in the survey questions. In contrast to our pre-registered protocol, we have not graphically presented the interview responses regarding predicted behaviour in response to flavour restrictions, intentions, and willingness to vape or use unflavoured e-liquids, as the responses were ambiguous in many cases, making quantification imprecise.

We used Interpretative Phenomenological Analysis (IPA) to analyse the qualitative data. IPA is a methodology in which the analyst takes an active role in interpreting how participants make sense of their social and personal world [45]. The most common IPA approach is to use transcripts from semi-structured interviews to identify common themes to explore the personal perceptions or accounts of an event [46]. In this analysis, two researchers analysed 50% of the data each by transcribing the recordings, reading the transcriptions, and making notes (i.e., coding the transcript). This process was repeated by a third researcher who resolved any disagreements about codes between the other researchers. The third researcher then compiled emerging themes by condensing the related notes into concise phrases which referred to a higher-level concept. The themes were then clustered into superordinate and subthemes by identifying conceptual similarities between them. Finally, we reviewed the themes in relation to the transcript notes to check that they suitably reflected the notes and were named appropriately.

## Results

### Participant characteristics and baseline data

Participants who smoked and participants who vaped were similar in age (ranging from 19 to 62 years), gender, ethnicity, number of cigarettes smoked (currently or in the past) and smoking history (Table 1). Participants who vaped reported using a range of e-liquid nicotine strengths (some reported using more than one), but all participants used less than 19 mg/ml (in line with UK regulations). All participants used fruit flavoured e-liquids, but some participants also reported using other flavours (either in the survey or during the interview) such as “pastry flavours”. Responses to this questionnaire item, such as "various berry flavours”, limited our ability to determine how many flavours or which specific flavours were used by each participant (Additional file 2: Table S5). Participants reported the average puff count (84 for participants who smoked and 83 for participants who vaped) and duration (8 min, 56 s for participants who smoked and 7 min, 23 s for participants who vaped) displayed on the e-cigarette device. Frequency of e-cigarette use can be difficult for people who vape to estimate [21] and at least one participant reported the number of puffs they took per day instead of the number of times they vaped per day.

**Table 1:** Participant characteristics and survey responses relating to a hypothetical flavour restriction

Variable	Participants who vaped (N = 12) N (%)	Participants who smoked (N = 12) N (%)
Female gender	7 (58%)	7 (58%)
Male gender	5 (42%)	5 (42%)
Black/African/Caribbean/Black British Ethnic group	0 (0%)	1 (8%)
White Ethnic group	12 (100%)	11 (92%)
Smoked daily for 6–12 months	1 (8%)	0 (0%)
Smoked daily for 1–2 years	1 (8%)	4 (33%)
Smoked daily for 2–5 years	1 (8%)	3 (25%)
Smoked daily for 5 + years	9 (75%)	5 (42%)
Wants to quit smoking		
Yes	–	1 (8%)
Maybe	–	9 (75%)
No	–	2 (17%)
Ever vaped	12 (100%)	8 (67%)
Vaped for 3–6 months	6 (50%)	–
Vaped for 6–12 months	5 (42%)	–
Vaped for 1–2 years	1 (8%)	–
Nicotine strength: 1–3 mg/ml	3 (25%)	–
Nicotine strength: 4–6 mg/ml	2 (17%)	–
Nicotine strength: 7–9 mg/ml	2 (17%)	–
Nicotine strength: 10–12 mg/ml	3 (25%)	–
Nicotine strength: 13–15 mg/ml	1 (8%)	–
Nicotine strength: 15–18 mg/ml	1 (8%)	–
E-liquid flavour used (fruit)	12 (100%)	–
E-liquid flavour used (other)	2 (17%)	–
	Mean (SD)	Mean (SD)
Age (years)	31 (11)	27 (12)
Cigarettes smoked per day	13 (5)*	13 (6)
Readiness to quit score	–	4 (2)
Times vaped per day	24 (17)	–

Responses recorded prior to a 4-h trial of unflavoured e-liquid. Only the options selected by participants are displayed in the table. Some questions were not asked to either people who vaped or people who smoked in the Qualtrics survey; a dash (-) indicates this question was not asked. The readiness to quit ladder ranges from 1 (I have decided not to quit smoking for my lifetime, I have no interest in quitting) to 10 (I have quit smoking) [1]. *Cigarettes smoked per day prior to stopping smoking

**Fig. 1:**
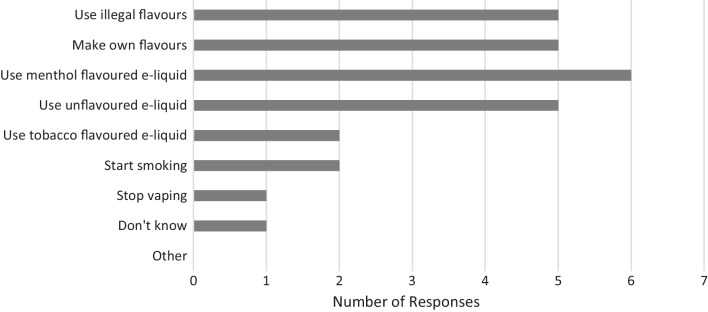
Actions that people who previously smoked and subsequently vaped would take if there was an e-liquid flavour restriction (N = 12). Participants responded to multiple choice questions relating to a hypothetical flavour restriction in which only unflavoured, tobacco flavoured, and menthol flavoured e-liquids remained on the market (recorded at baseline)

**Fig. 2:**
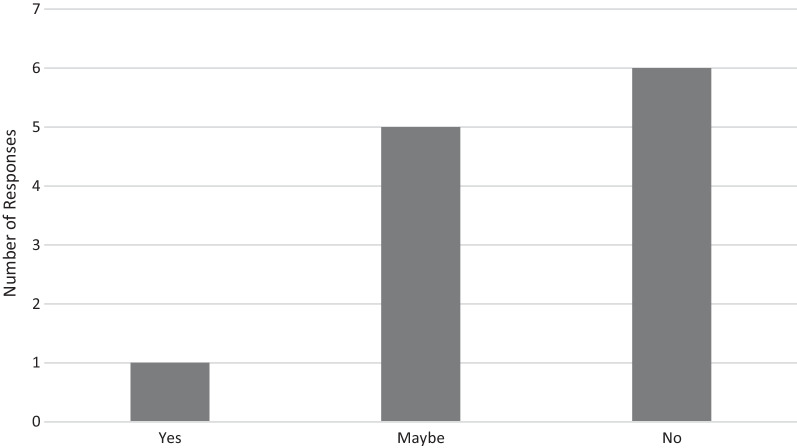
Perceived success of participants who used a vape to stop smoking if they had used unflavoured e-liquid to stop smoking instead (N = 12). Participants responded to the question: "If you had used an unflavoured e-liquid when you quit smoking, do you think you would have successfully quit?" (recorded at baseline)

**Fig. 3:**
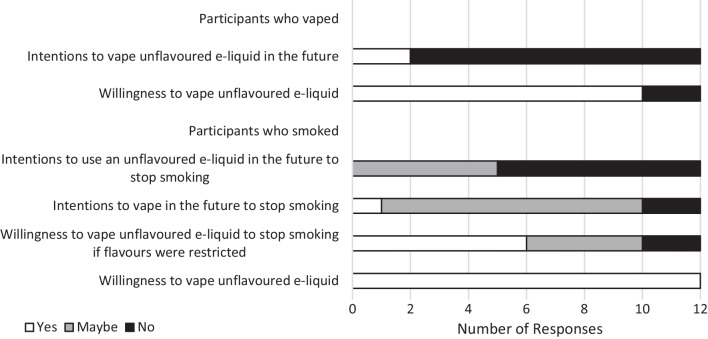
Participants’ willingness and intentions to vape or vape unflavoured e-liquid. Response options were “Yes” or ‘No”, with no “Maybe” option in the survey for participants who used to smoke and subsequently vaped. Responses were recorded at baseline

Baseline quantitative data regarding predicted behaviour in response to flavour restrictions, intentions, and willingness to vape or use unflavoured e-liquids are presented in Figs. 1, 2, 3.

### Themes

We identified six superordinate themes through IPA (Table 2). The ‘intentions and motivations to stop smoking and/or use e-cigarettes’ theme provides insights into the participants’ past and/or future likelihood of smoking and vaping irrespective of a flavour restriction. As this provides context but does not answer our research question, it can be found in Additional file 1, Sect. 2.1. Other superordinate themes are discussed below except for subthemes which were infrequently raised or ambiguously linked to the superordinate theme ‘other drivers of vaping behaviour are more important than flavours’ (Additional file 1, Sect. 2.2). For clarity, we indicate which participants vape and which smoke with a ‘V’ or ‘S’, respectively, at the end of their participant number.

**Table 2:** Superordinate themes and subthemes of participant opinions, beliefs, and intentions

Superordinate theme	Subthemes
Intentions and motivations to stop smoking and/or use e-cigarettes	Stopping smoking Barriers to stopping smoking Motivation to stop smoking Motivation to use e-cigarettes E-cigarettes are for smoking cessation Trial of others’ e-cigarettes
Sensations and experience of using unflavoured e-liquid	Throat hit and harshness of using unflavoured e-liquid Unflavoured e-liquid satisfied cravings for nicotine Unflavoured e-liquid satisfied cravings for cigarettes Similarity to smoking Expectations of unflavoured e-liquid Opinion of unflavoured e-liquid Potential use of unflavoured e-liquid to stop vaping Ambivalence towards unflavoured e-liquid Amount of unflavoured e-liquid used
Taste of unflavoured e-liquid and flavour preferences	Appeal of flavours Flavours are unappealing Trial of other e-liquid flavours Dislike of specific flavours Flavour preferences Unflavoured e-liquid is flavourless Unflavoured e-liquid is not flavourless
Personal impact of a hypothetical flavour ban	A flavour ban would affect likelihood of quitting smoking or relapse A flavour ban would not affect likelihood of quitting smoking or relapse A flavour ban is not a good idea Flavour preferences in the event of a flavour ban Amount of e-liquid would use if there was a flavour ban
Impact of a hypothetical flavour ban on others	A flavour ban is a good idea A flavour ban is not a good idea Alternatives to smoking should be promoted not discouraged Flavours are attractive to young people and people who do not smoke Opinion of the gateway hypothesis Comparing restrictions to other products and activities Flavour preferences of others
Other drivers of vaping behaviour are more important than flavours	People do not use e-cigarettes just because of the flavours Effectiveness of the e-cigarette Nicotine is more important than flavours Ease of e-cigarette use Health and addiction concerns

### Sensations and experience of using unflavoured e-liquid

In this theme, participants described the ‘sensations and experience of using unflavoured e-liquid’. Participants often described their experience of using the unflavoured e-liquid in terms of the harshness and throat hit, with people who vaped usually describing a harsher experience than their usual product and people who smoked describing a lesser throat hit compared to smoking. P015V said “I thought it felt quite harsh” and P020V stated, “it was just like nothing, except like just the harsh feeling of the smoke itself.” In contrast, P025S said, “There was no coughing or that harshness which is something you have when you have a cigarette” and P022S said “it didn’t really give that hit on the erm… throat that the cigarettes normally give you.”

The majority of participants who smoked thought the unflavoured e-liquid satisfied their cravings for cigarettes or nicotine but for others, it did not satisfy other elements of cigarette enjoyment. P001S said, “it gave me nicotine, and like I didn’t crave a smoke at all” and P012S said, “it definitely eliminated my cravings for cigarettes because I was constantly reaching for [the e-cigarette].” P021S said “I’d definitely say it hit the […] nicotine craving, but probably not the sort of unconscious pleasure I get from sort of having a cigarette compared to a vape.” P002S thought that the remaining craving was for the “ritual of smoking as opposed to like just the nicotine side of it.” Some participants who vaped also stated that the unflavoured e-liquid satisfied their cravings for nicotine, but they did not enjoy the experience. When P014V was asked “did you enjoy using it?”, they responded, “I kind of did in a way, because […] it’s satisfying a craving, the craving for nicotine, […] but the thing that I disliked really was the fact that it’s unflavoured”. P009V said the unflavoured e-liquid “quelled the, the want for […] nicotine generally, but I, I didn’t enjoy the experience, which I would normally with the flavoured stuff.”

Some participants commented on the similarities between smoking and using the unflavoured e-liquid. Most participants who smoked made general comparisons about vaping which were not specific to the unflavoured e-liquids, but P022S stated, “in terms of taste I cannot say that I’ve seen any noticeable difference [between using unflavoured e-liquid and smoking], which is good because it can serve as a replacement.” P002S said “I’ve tried the tobacco [e-liquids] and they’re not, they’re not quite like tobacco […] there’s more of a resemblance in the unflavoured one.” P020V was less positive about the similarity and said, “obviously it is unflavoured, and it, it didn’t have a flavour, but at the same time, it did, in the sense of, it’s just, it was just harsh, and dry […] like smoking”. P023S said, “I know it’s designed to simulate tobacco, which it does sort of an alright job of, but it’s basically the same but slightly more horrible.”

Participants generally had no expectations or negative expectations prior to using the unflavoured e-liquid. Where participants had no expectations, it was usually because they had not heard of unflavoured e-liquids. P015V stated, “I didn’t actually know they existed, I thought it was just tobacco, menthol and the other flavours”, and “I didn’t really have an opinion I just thought, ‘this is going to be horrible’”. Among those who had negative opinions prior to using the unflavoured e-liquid, some were pleasantly surprised; P014V said, “I felt like I was gonna be ripping me hair out for four hours, [because] I haven’t got my normal berry vape with me, but it wasn’t actually that bad really”. Others had their negative expectations confirmed, like P017V: “I expected it to be pretty bad, and it was pretty bad” and “it can’t even stand in the shadow of what I normally vape.” Participants who smoked tended not to have strong expectations about the unflavoured e-liquid prior to trying it, but some were positive after. For example, P003S admitted “I didn’t think I was going to enjoy it, and I didn’t see the point in having an unflavoured one, but actually […] I didn’t mind it and it was, it was quite nice.” P021S said, “unflavoured liquid would be the way I would go if I decided to take up vaping in the future”. Some participants who smoked were ambivalent after trying unflavoured e-liquids. P011S shared, “I thought it was going to taste worse than it did. […] I didn’t have any like ‘wow this is amazing’ either.” Some participants who smoked had negative opinions of the unflavoured e-liquid, like P023S who described it as “a flavour that you’d rather not have”.

There were mixed opinions among participants who vaped. P004V and P006V found it “worse” than they expected and P020V’s ambivalent opinion changed to it being “vile” after trying the e-liquid. P020V said, “you might as well just not have anything or […] not quit smoking.” P017V said “unflavoured’s pretty grim” and “it’s like having unflavoured toast, you’re not just gonna have like a bit of toast in the morning with nothing on it, are you? Like you probably could, you wouldn’t enjoy it, but, if you can, chuck a bit of flavour on there.” P019V said “It’s like drinking water instead of squash” and “I’m not sure I would choose the unflavoured again” because they preferred “fruity.” Some participants who vaped were more positive, for example P013V said, “I wouldn’t of chose it, but […] it’s alright” and “I would definitely use it again”. Some participants who vaped were not overly positive but said they “could probably get used to it” (P016V) or “could probably firm it out and get used to it after a week or two but I wouldn’t enjoy myself for that week” (P017V).

Some participants who vaped commented on the use of unflavoured e-liquids to stop vaping or using nicotine products entirely. If P006V were using e-cigarettes to “withdraw from nicotine completely then […] I could see like a chemist giving [unflavoured e-liquid] out for example, because there’s not much enjoyment to it.” P015V said, “I’d say it’s quite likely [I would use unflavoured e-liquid again] because I don’t think I’m ever gonna get off vaping completely if I’m having things that are really nice” and P009V said “if I wanted to stop vaping maybe I should stop making it taste like Eton mess [a dessert containing cream, meringue and fruit.]” P020V said unflavoured e-liquid “is a good way to maybe completely get rid of all type of nicotine products because it would put you off in a way […] but, I mean I genuinely would rather have a cigarette.” Not all participants who vaped said that unflavoured e-liquids are useful for stopping vaping. P014V said “unflavoured liquid wouldn’t really help me in any way to, to stop vaping because I’m still gonna crave that nicotine hit, you know? If I wanted to stop vaping, I would have to use patches.” P018V said, “if I was to wean myself off vaping, I’d probably try and do it with one with maybe like no nicotine in, […] but I would still go for a flavoured one, I wouldn’t want the unflavoured.”

Many participants who vaped reported that they used their e-cigarette less than usual while using the unflavoured e-liquid. P004V thought during the 4-h trial period they “vaped like nowhere near as much as I usually would in this time. Just because I think it wasn’t like as much fun” and P006V said, “I don’t really wanna use it.” P015V, P017V, P018V, P019V and P020V all thought that they vaped less than usual too, but P016V vaped “about the same” and P014V thought they vaped “a lot more” which they reported was “because [of] the lack of flavour, I wasn’t really getting the full satisfaction that I normally get, so I was constantly on it.”

### Taste of unflavoured e-liquid and flavour preferences

Discussions of the taste of the unflavoured e-liquid and participants’ own preferences for specific flavours versus preferences among youth and those who do not smoke formed a superordinate theme. There was variety in the flavours that participants disliked or preferred and many described trialling different flavours when considering using e-cigarettes and during their transition from smoking to vaping. P010S said, “they’ve got all these flavours so let’s try them”, and P007V described a process of “trial and error” in finding the right flavour for them. Many participants who vaped preferred “primarily the fruit ones” (P004V), including one participant who initially assumed they “wouldn’t have flavoured liquids, because it doesn’t really mimic smoking” (P015V).

After trying the unflavoured e-liquid, some preferred it to flavours such as menthol or fruit/sweet flavours, but most preferred flavours. There were individual differences in which flavour participants stated they would choose if only tobacco, menthol or unflavoured e-liquids remained on the market; some stated tobacco, some stated menthol, and some stated unflavoured, but some, like P004V “would prefer to use cigarettes over [unflavoured e-liquid]”.

When prompted about the taste and flavour of the unflavoured e-liquid, some participants described it as “unflavoured”, “plain”, like “water” whereas some described a “metallic” or “burnt” taste, and some reported a hint of “sweetness”.

Many participants found the variety of flavours appealing when they initially decided to vape, and some participants who smoked also discussed the appeal. P014V found the variety of flavours available “extremely appealing”, P015V found “the variety quite helpful”, and P017V said “there’s like so many different flavours and there’s like you can have anything you want pretty much” which “hundred percent” influenced their decision to vape and made switching from smoking to vaping a “smoother transition”. The appeal of flavours persuaded P013V to make the switch due to the “nice smells”. P024S thought they “would prefer vaping if it was flavoured.” Some participants thought flavours also appealed to children and people who do not smoke. P016V believed “like the strawberry cheesecake ones […] they’d definitely appeal to younger kids. I reckon that’s probably what they would smoke if they did smoke them” and P020V thought “a hundred percent, yeah, I think it does create an appeal [to people who do not smoke]”. Not all participants found vaping appealing (e.g., P002S found flavours “too sweet”) and not all participants said that flavours appealed to children either. P025S disagreed with the argument that “because they’re young, they must go for fruit stuff” and said “it’s more the adults that go for it, strangely enough, because it reminds them of their childhood.”

### Personal impact of a hypothetical flavour ban (excluding tobacco and menthol)

Some participants, particularly those who smoked, were confident a ban on flavoured e-liquids (whereby unflavoured, tobacco flavoured, and menthol flavoured e-liquids would remain on the market) would not personally affect them. P011S said, “for people like me, you know smokers, it wouldn’t bother me too much”. P008S said, “I could still erm use them to quit” but thought “it might not be as encouraging.” There were some participants who vaped, particularly those who enjoyed using the unflavoured e-liquid, who “would definitely keep to using [unflavoured e-liquid], because it’s not harsh” (P013V). Some participants who vaped, like P016V, did not feel their quit attempt would have been hampered in this scenario: “I do think I would [have successfully quit using an e-cigarette], because you still get the hit off it […] I think if I hadn’t tried flavoured, I wouldn’t know any different.” Some participants’ confidence that they would not be affected by flavour restrictions seemed to stem from their commitment to never smoke again: “I’m definitely out of that habit, I never, never want to go back to that habit again” (P014V). Some felt that “enough time has passed” since quitting that they may be able to cope with the change (P007V). One participant discussed continuing to make their own flavours if “ingredients were still available” (P007V).

Some participants reported that they would be negatively impacted by flavour restrictions. Some who smoked thought that they would not attempt to quit: “if it was only unflavoured, I probably wouldn’t bother” (P003S). A few participants who vaped suggested that they may have never tried to quit smoking in this scenario, for example P006V said “I probably wouldn’t of bothered stopping smoking if that had been the case.” Whereas some said they would have tried vaping but “might not […] have been as successful at stopping smoking” (P014V). Some participants who vaped thought they would return to smoking like P019V who would try “whatever was available… for a while, but then I, I think I’d probably go back to smoking”. Some participants who vaped “would just look towards quitting” nicotine products entirely (P017V). A few participants who vaped thought that a flavour restriction would cause them to vape less, like P004V, who did not think they “would vape nearly as much”. Some participants who vaped reported that they would try to access flavoured e-liquids illegally like P015V, who said “I would try and get it elsewhere if I was really motivated […] depends on how far along I was that I wanna quit, but at this stage now, I would probably get it elsewhere somehow.”

Although some participants supported a restriction on flavoured e-liquids in the UK, none stated personal reasons for this. Many stated personal reasons for a ban in the UK being a “bad idea”. When asked if flavours should be banned in the UK, P018V said, “because I vape and I vape the flavoured ones, I would say no.” Some thought we should not “be attacking what’s been like a really good way to get people to quit smoking and use something healthier” (P002S). Others thought that people “should have a choice. It’s up to people to choose to do possibly unhealthy things if they want to” (P024S). P009V said, “selfishly I don’t want it to happen because I like Eton mess”, which echoed comments from other participants who did not want their favourite flavours to be removed. Some participants would only be in support of a ban if “a causal link has been established between [flavourings and] negative health outcomes” (P007V). P016V thought “you could find it if you wanted it” even if the flavours were banned. Many suggested that they would be more supportive of other restrictions and regulations over banning flavours except from menthol and tobacco; instead of “command and control, […] monitoring it and regulating it” (P011S). The alternative policies suggested by the participants included adding age restrictions, stricter age verification, reducing appeal of packaging, restricting only some flavours which most appeal to youth, and better marketing restrictions.

### Impact of a hypothetical flavour ban on others (excluding tobacco and menthol)

Although participants did not see a personal benefit resulting from restricting non-tobacco flavoured e-liquids, some participants thought the hypothetical favour ban would benefit youth as they thought some flavours may appeal to children. P015V thought some flavours should be banned because “strawberry laces, and things like that, that’s just screaming to children, to me”. P003S thought: “I see a lot of young people vaping. So, I do think that removing them from the market probably would help that, and I don’t think any kid would think it’s very cool to be puffing on an unflavoured liquid.” P004V spoke of friends who “didn’t really smoke and then they started vaping like a lot just because it tastes nice and like it’s something to fiddle with and it gives you like a niccy rush and like serotonin rush” and thought a flavour ban “would definitely be a good idea.” P021S thought that “popular vape companies are aware of that these sort of e-liquids are attractive for young children” and thought “it should be banned if it’s something that’s getting out of control.”

Many participants thought that the benefit to youth did not outweigh the benefit to adults who smoke or vape who could use these flavours to refrain from smoking, and some thought less harmful alternatives to smoking should be promoted rather than discouraged. P002S said “the benefits [of flavour availability] probably outweigh the risks” and that vaping has “helped a lot of people quit and [a number of people] demonstrably higher than sort of any number you could conjure up of people who have taken up smoking off the back of sort of starting with flavoured e-liquid from when they were young.” P019V wondered “are more people smoking now, [in countries which have restricted flavours], because they haven’t got the option to vape? Or what they want to vape, the flavours they want to vape?” P007V thought “it wouldn’t be helpful” and “it would slow down the rate of people that are quitting smoking.” Although they “wouldn’t want young people to start to vape” they would “want people who smoke to, to do whatever it takes to stop smoking and […] if they’re like me, then vaping’s been the only thing […] that’s made that possible, so I would want to give everyone else the opportunity to do that too.” P011S thought “stopping young people from smoking is great for future generations” but “the focus should also be on current smokers, to stop them from smoking and try and find an alternative means.” P015V thought that restricting all flavours could lead to increased “criminality” and highlighted that any new policy “needs to be safe, ‘cause if they ban it completely then they’ll just open it up for things to become very unsafe” for people who use e-cigarettes.

Not all participants said that the hypothetical restrictions would be beneficial to youth either, primarily because they thought it would be ineffective in preventing vaping among youth. P023S thought the more you tell young people “they can’t do something, the more they’re likely to do it if anything.” P024S said, “if kids wanna do something a bit rebellious or something they’re not meant to do, they’re gonna do it anyway.” They said when they “first started smoking when I was 13… I just did it, I didn’t go for the flavoured products, I just used the regular ones because my friends were doing it.” P025S thought that youth today are “social media’s [generation] not the candyfloss generation. So, I don’t think it matters whether you stop fruit.” They thought if we keep associating flavours like “bubblegum and candyfloss with just kids, then that works for the younger kids in that generation of like toddlers […] but, I think for the younger teens… most of the stuff the parents think [they haven’t] tried, they probably have.” They said, “I don’t think if you ban it, it will stop anything. They will just go for the next thing that’s available.” P010S said, “you’re not going to stop young people from experimenting with tobacco or anything, so, […] they would probably find something that… had a flavour… that… in my guess would be some weed.”

Some participants thought that restrictions would be ineffective, particularly if the aim was to prevent youth from smoking, as they were sceptical about the gateway hypothesis. P002S was “sort of sceptical of the sort of like gateway argument” because “the flavour of flavoured e-liquid is so drastically different to the actual flavour of tobacco.” They said, “if the only options are tobacco flavoured, then they’re more likely to find the adjustment to… actually going onto smoking” easier. P019V thought “vaping is definitely more appealing than smoking, but then I can’t imagine why people would start smoking after trying vaping”. P011S said that it is hard to generalise, it is not that “they have one puff on this flavoured e-cigarette and that’s it, they’re hooked, they’re a smoker, and you know… it’s all going to go downhill from there. That’s… that’s not the case.” P008S said “it does the opposite” and they knew “a lot of people that it has helped […]. Some of them have quit [smoking].” The experience of P017V was more in line with the gateway hypothesis; they “didn’t really smoke” and tried vaping because it was “nicer”, “different”, “socially acceptable” and potentially less harmful than smoking. They then went through a “cycle of smoking, vaping” and were supportive of a ban on flavours to protect youth from smoking.

Some participants suggested alternative actions to take instead of restricting flavours. P021S suggested increasing awareness that the product is not for young people, but they thought a ban should only be considered “if it definitely would help kind of cut down the numbers of children vaping.” P022S suggested using social media to discourage youth vaping alongside other methods because youth vaping is “a really complex problem, hence it requires a complex solution.” Others thought we should use existing regulations for other products as a guide. P007V thought “regulations around smoking are more or less appropriate” and they “should be similar for vaping.” P017V compared vaping regulations to alcohol regulations: “when you buy alcohol and stuff and it’s delivered, they like check your ID at the door, rather than just handing over like a crate of wine to a ten-year-old.” They went on to compare vaping to gambling, “like betting on your phone, you have to go send your ID off, and your driving licence off and it has to be linked to your bank account, so, it’s definitely you. Why can similar things not be put in place for buying alcohol, tobacco products?”.

### Other drivers of vaping behaviour are more important than flavours

Participants identified other drivers of vaping behaviour they thought were more important than flavours in the appeal and use of e-cigarettes (with respect to themselves or others). P007V said, flavours did not impact the reason they started smoking so they “would be surprised if flavour alone was influencing young people deciding to start vaping.” The most common driver of vaping behaviour that participants thought was more important than flavours was nicotine. Like many others, P014V was “vaping to get the nicotine hit […] the flavours just a bonus really.” P025S said, “flavours isn’t the issue, because it’s the nicotine that’s the issue. […]. There’s flavours out there now, but… that’s not what’s causing the young people to try it.”

Health concerns (e.g., considering the comparative health risks and benefits of vaping compared to smoking) were prominent in the decision-making process of whether to use e-cigarettes or not for some participants. P001S was wary about e-cigarette use and “read loads of articles saying that they like […] don’t really understand them” which “scared” them. P020V said “there’s still risks to vaping, but nowhere near as bad as smoking.” P013V vaped because they “just thought it’s got to be healthier for you than smoking.” Some stated that they would only stop using flavoured products or vaping if “some like scientific research was done, and it turned out that the flavoured ones are very, very bad for you. You know, as bad as smoking and they can damage the lungs whereas the unflavoured ones, is very minimal damage” (P014V). Some participants who smoked mentioned their concerns about the potential addictiveness of e-cigarettes which discouraged them from using e-cigarettes. For example, P012S was wary that “if I did put a flavour in here, my consumption would very much, like, increase from it” because the unflavoured e-liquid was “not that pleasant to do, but it still has the addictive quality. Whereas if it was pleasant to do, and had the addictive quality” they would use it more. Some thought health concerns could feed into the decisions young people make too. P010S thought “young people these days […] tend to be a lot healthier” and would therefore be more wary of trying e-cigarettes. Some thought that the lesser health risks were reason to not be concerned if youth vaped instead of smoked. P002S said, “I don’t believe that there’s a lot of people that are picking up sort of e-cigarettes just because it’s flavoured” and if they were “there’s not much reason to believe it’s […] that harmful to health and if it is then it’s certainly most likely less harmful than if they were to pick up smoking.”

More often, however, participants thought the trendiness of e-cigarettes and peer pressure were the biggest drivers influencing e-cigarette use among young people. P001S suggested “it probably is just because they are sweet and then they look cooler or something.” P019V proposed people who do not smoke vape because “it’s like a peer pressure thing” and P025S said: “this is the social media generation, so, everything’s a hype” so young people will do “whatever they see the celebrities do” and “the celebrities don’t look like they’re stopping anytime soon. So, it’s the new thing.” P022S recalled that after watching films and TV, “seeing imagery of people smoking […] to some extent just subconsciously I started mimicking them in some way and I haven’t really seen anyone vaping.” P023S suggested “there should be [senior male celebrity] on the BBC smoking flavoured vapes to make it as uncool as possible” to discourage youth use. Other drivers of vaping behaviour discussed included the ease of vaping, cost of vaping, the social acceptability, the effectiveness of the device, to get breaks at work, behavioural aspects, and peer pressure (Additional file 1, Sect. 2.2).

## Discussion

This study provides an insight into the potential impact of e-liquid flavour restrictions in the UK. At a time when there is considerable pressure on the UK government to address the rise in e-cigarette use among young people, this study indicates the impact of a flavour ban from the perspectives of people who have used and could use e-cigarettes to quit smoking. When making policy decisions, it is important to consider evidence such as this to avoid negative consequences and increase the likelihood of the policy reducing population-level harm. We found six superordinate themes which centred around the intentions and motivations to stop smoking and vaping, the sensations and experience of vaping unflavoured e-liquid, the taste of unflavoured e-liquid and flavour preferences, the negative impact of flavour restrictions on the participants, the positive and negative impact of flavour restrictions on others, and other drivers of behaviour being more important than flavours.

Our findings are consistent with previous evidence suggesting that the experiential aspects of e-liquids are important and could influence behaviour. For example, the harshness of the unflavoured e-liquid was discussed by participants. Previous research has shown that harshness of e-liquids is a quality that is disliked by people who use e-cigarettes [29], but throat hits can be pleasant or unpleasant for people who smoke, and finding the optimal throat hit is associated with increased desire to quit smoking using e-cigarettes [16]. Our results suggest unflavoured e-liquids may be too harsh for people who use e-cigarette who have adjusted to less harsh, flavoured e-liquids and not harsh enough for some people who smoke who enjoy a strong throat hit. The unflavoured e-liquid was sufficient to satisfy some participants’ cravings for nicotine and cigarettes, as we have found previously [18], but they were not as enjoyable for some who currently use flavoured e-liquids. Some participants felt that the switch from smoking to vaping was made easier because of the similarities between the two behaviours, but the desire to mimic cigarettes may decrease over time. Some participants who smoked had tried vaping but stopped because the flavour differed from smoking, whereas those who continued to vape seemed to prefer there to be a difference in flavour. When initially quitting, similarity to smoking and tobacco flavour may be important, but Farsalinos et al. [19] found that flavour variability is very important to people who use e-cigarettes who have successfully stopped smoking. In line with our findings, other research has shown people who smoke report using a preferred flavour when starting to vape, which may take some trial and error to find, but some people continue to seek variety [8] and many people who vape regularly use multiple flavours [17, 43, 44]. Our survey results support this, with most participants reporting using a variety of flavours rather than one specific flavour (Additional file 2: Table S5). Some participants who vaped claimed they vaped less than they usually would when using the unflavoured e-liquid provided. Infrequent vaping is associated with greater risk of relapsing to smoking compared to frequent vaping [10], so changes in frequency of use due to flavour restrictions could impact the likelihood of relapse to smoking.

As suggested by many of the participants, current evidence suggests that flavours other than tobacco and menthol appeal to non-smoking young people as well as adults who smoke and/or vape [30, 35, 36], but the participants raised many potential issues that could arise from a restriction on e-liquid flavours which could ultimately result in increased population-level harm. One potential issue raised was that the restrictions may lead more people to start or continue to smoke cigarettes. Consistent with this perception, flavour restrictions in San Francisco were reportedly followed by reductions in e-cigarette use but increases in smoking among young adults [22, 54]. Buckell et al. [12] found that while flavour restrictions could reduce choice of e-cigarettes by 11% they could also increase choice of cigarettes by 8% among people who smoke or have recently quit. Another potential issue was that some thought they would make their own e-liquids or obtain them illegally, which reflects what occurred in Finland, where 43% of people who vaped in the year after flavour restrictions were introduced used banned flavours [42]. The use of illicit and adulterated e-cigarette products, and products from informal sources can expose people to additional harms: 2,807 people were hospitalised, and 68 people were killed during the e-cigarette and vaping associated lung injury (EVALI) outbreak (July 2019 to February 2020), when vitamin E acetate was added to e-cigarettes to vape Delta-9-tetrahydrocannabinol [THC] instead of nicotine [9]. For these reasons, some participants did not think flavours being restricted in the UK was a good idea, in line with survey data from the International Tobacco Control (ITC) study which found that a ban on non-tobacco flavours would be strongly opposed by more than 81% of people who vape [26].

Despite the potential issues raised, some participants were still supportive of flavour restrictions to discourage youth from initiating vaping. In line with these participants’ opinions, after flavour restrictions were introduced in Finland, e-cigarette use has remained low among 15- to 69-year-olds [42], and the prevalence of e-cigarette use among young adults has decreased among young adults since the ban in San Francisco and other US states [13, 54]. Other participants suggested that alternative measures could be more effective, and that there are other more important drivers of vaping behaviour than flavours, such as health concerns and trendiness. The alternative measures suggested, such as increased age restrictions and stricter marketing regulations, have been implemented in other countries with varying success [53]. Patel et al. [37] found 85% of US adults who vape cited health and smoking cessation as a reason for using e-cigarettes, 57% cited convenience, and 34% cited flavouring. Although younger adults (18–24 years) were more likely to cite flavours (46%) than the older adults, they were more likely to report health/cessation (73%) and convenience (55%) as reasons for use [37]. In UK adults, a reduction in beliefs that e-cigarettes are less harmful than combustible cigarettes was associated with a decrease in the prevalence of e-cigarette use. Trendiness has also been reported to influence youth use both in the UK and US in qualitative interviews with youth [11, 49].

This study allowed us to explore various experiences and opinions from people of different ages (19–62 years) and people with different smoking and vaping histories, however, the participants were predominantly White. We acknowledged that few people who vape in the UK use unflavoured e-liquids, so participants tried an unflavoured e-liquid before commenting on flavour restrictions that would exclude unflavoured e-liquids.

Although participants were allowed to trial the unflavoured e-liquid for 4 h, taste profiles can change after stopping smoking, so the findings may have been impacted by the short trial period as well as the lack of tobacco and menthol e-liquid provision, the use of unfamiliar devices, and potential device malfunctions. Participants who smoked were only offered unflavoured e-liquid and not flavoured e-liquid and they did not receive a live demonstration or live advice on how to use the e-cigarette, which could have influenced their responses. Although eight out of twelve of these participants had tried vaping (and had likely experienced flavoured e-liquids before), the inhalation processes can considerably differ between smoking and vaping with increased experience [20], so participants may have had a more positive experience with further guidance on how to use the product. These findings are reflective of the participants subjective estimation of the impact of a hypothetical restriction rather than an objective observation of the impact, and the research was conducted before the UK government announced plans to restrict flavours. Future research could explore the potential impact of other restrictions which have been announced by the UK government, such as restricting the sale of disposable e-cigarettes, to identify which regulations may have the least negative impact on people who currently smoke and vape. The impact of unflavoured versus flavoured e-liquids on vaping frequency and smoking cessation could also be explored.

## Conclusions

In conclusion, there are differences in how individuals who smoke or vape perceived they may be impacted by e-liquid restrictions in the UK. Some believed they would be unaffected as they would use unrestricted flavours or continue to smoke, but some felt they would be at greater risk of relapsing to smoking or continuing smoking rather than quitting with an e-cigarette. Most participants seemed to support the prevention of young people from starting to vape, but they had differing opinions on whether restricting flavours would be an effective method to discourage youth vaping. These results reflect participant perceptions of the impact of a flavour ban, but actual behaviour in the event of a restriction may differ. Nevertheless, the subthemes identified here could be used to guide further research into the impact of flavour restrictions which could be used to aid policy decisions to reduce harm related to smoking and vaping.

### Supplementary Information

**Additional file 1. **contains supplemental texts relating to methods (Sect. 1) and supplementary themes (Sect. 2).

**Additional file 2.** contains Supplementary Tables S1–S5.

## Data Availability

Data are available at the University of Bristol data repository, data.bris, at https://doi.org/10.5523/bris.1hr9weuiqmiq52344a0wczg00i.

## Abbreviations

BBC: British Broadcasting Company
CPD: Cigarettes per day
E-cigarettes: Electronic Cigarettes
IPA: Interpretative Phenomenological Analysis
ITC: International Tobacco Control
PG: Propylene glycol
TARG: Tobacco and Alcohol Research Group
UK: United Kingdom
US: United States
USA: United States of America
USB: Universal Serial Bus
VG: Vegetable glycerin
