# Supplementary material for: Exploring the opinions and potential impact of unflavoured e-liquid on smoking cessation among people who smoke and smoking relapse among people who previously smoked and now use e-cigarettes: findings from a UK-based mixed methods study
Source: Harm Reduct J. 2024 May 3;21:90. doi: 10.1186/s12954-024-01003-z (PMC11067290; doi:10.1186/s12954-024-01003-z)
Supplement: Supplementary file 1 — Additional file 1. contains supplemental texts relating to methods (Sect. 1) and supplementary themes (Sect. 2). [file 12954_2024_1003_MOESM1_ESM.docx]

**Additional File 1 for** **“Exploring the opinions and potential impact of unflavoured e-liquid on smoking cessation among people who smoke and smoking relapse among people who previously smoked and now use e-cigarettes: Findings from a UK-based mixed methods study”**

**1.1 Inclusion and exclusion criteria**

The participants were able to give informed consent, able to attend two online study sessions and had not had (or been in contact with anyone who had) flu-like or coronavirus symptoms in the 10 days prior to the study. All participants had to be willing to try an e-liquid and e-cigarette and self-administer a urine test (all provided by the research team) to check for cotinine (a highly specific biomarker for nicotine) and pregnancy (if female). Those with known allergies to the e-liquid ingredients, current or past physical or significant psychiatric illness, uncorrected visual or hearing problems, current sensory problems affecting sense of smell or taste, and those who were pregnant, breastfeeding or planning to become pregnant during the study period were excluded from participation in the study.

**1.2 Assignment/choice of e-liquid**

The smoking heaviness of participants who reported smoking between 5-19 cigarettes per day (CPD) was classed as moderate and these participants received an e-liquid nicotine strength of 1.0% (10 mg/ml). The smoking heaviness of participants who reported smoking ≥ 20 CPD was classed as heavy and these participants received an e-liquid nicotine strength of 1.8% (18 mg/ml). 1.0% (10 mg/ml) represents the average nicotine strength of e-liquid purchased by a vape shop customer (Smets, Baeyens, Chaumont, Adriaens, & Van Gucht, 2019), and the higher dose accommodates the needs of people who smoked more heavily. Participants who vaped (who are more accustomed to what nicotine concentration suits their needs) were given the option of which e-liquid nicotine strength they would prefer.

**2.1 Intentions and motivations to stop smoking and/or use e-cigarettes**

Among the participants in the study who smoked, 8 out of 12 stated having tried to stop smoking in the past. Four participants reported successful past quit attempts like P024S: “I started smoking when I was quite young, I was like 13, so I quit when I was 16”. One reported having not tried very hard to quit: “I mean I had the [smoking cessation] stuff, but I didn’t really give it a longitude” (P025S). Two participants referred to cutting down their smoking as a quit attempt but did not try to stop completely: “I’ve just tried cutting down in the past, I’ve never tried to quit completely” (P021S). Of the participants who had previously tried to stop, all but P001S had tried e-cigarettes before, four had used e-cigarette out of curiosity or “just out of interest” (P024S), and three participants had used e-cigarettes regularly in the past either alongside smoking (P003S, P025S) or to replace smoking (P023S).

Among the four participants who had not previously stopped smoking before, most reported having no desire to quit because they liked/enjoyed smoking or because they were unconcerned by health effects. For example, P008S stated, “I will eventually quit sometime, but at the moment I’m young and healthy”, and P022S said, “there just isn’t any serious intentions [to quit] just because I really enjoy smoking”. Of the participants who had not tried to stop smoking, two had tried e-cigarettes before (P011S, P022S) and two had not (P008S, P012S). P011S said “I never really like properly used it”, and P022S had only had “a couple of puffs”.

Many participants (both those who smoked and used to smoke) described barriers to stopping smoking which included availability of cigarettes, being in social situations, and being under the influence of drugs (particularly alcohol). P023S said, “I got out [of a smoke-free prison] after two months and I wanted the first cigarette I could get” and P024S said, “I quit when I was 16 and when I was 18 it was obviously a lot easier for me to access tobacco products. So, I just sort of started again”. One participant even pre-empted social settings with alcohol acting as a barrier and relapsed to smoking in anticipation: “…as soon as it was announced that [COVID-19] lockdown was lifting and the pubs were reopening and I knew I was just going to be in the pubs all summer, I just thought I’d start [smoking] again because I knew I would eventually.” (P003S). Similarly, P017V said, “I’d never rule myself out from smoking again […] yeah, long term I don’t see myself everyday waking up and having a cigarette or vaping or whatever, it will just be more of a social thing.”

Current and past motivations to stop smoking varied among participants. Some participants who smoked were not motivated to stop. P012S was “not really that bothered [about quitting smoking] because… erm… I know that like lung capacity is recoverable.” However, health was an important motivation to quit for some; P020V switched from cigarettes to e-cigarettes for the “health benefits, even though there are still risks” and P021S wanted to quit smoking because “it’s not very good for you.” Other factors influencing e-cigarette use included money, COVID-19 and influence from friends and family.

As previously discussed, some participants who smoked were motivated to use e-cigarettes to stop smoking, whereas some used e-cigarettes out of curiosity. Both participants who smoked and previously smoked but now vape discussed the purpose of e-cigarettes being for smoking cessation. P012S said, “I kind of see [e-cigarettes] as something you would use to quit smoking” and P015V said, “I always saw vaping as something to try and help people get off cigarettes.” Participants also stated that they had tried other people’s e-cigarettes and e-liquids. For example, P001S said, “I do try other people’s [e-cigarettes] sometimes” and P004V said, “I knew like I’d liked it because I’d tried my friends vapes.”

**2.2 Other factors are more important than flavours**

In addition to the factors discussed in the main text, participants also discussed the ease of vaping, cost of vaping, the social acceptability, the effectiveness of the device, vaping to get breaks at work, behavioural aspects, and peer pressure. These factors were either less commonly reported or were less overtly stated as more important rather than additionally important to people who used the e-cigarette provided (e.g., some participants heavily focussed on the ease or effectiveness of the device when they were asked about the unflavoured e-liquid but did not overtly state that this was more important to them than the flavour).

How easy it is to use e-cigarettes was commonly discussed. This was often discussed among participants who smoked in response to using the device provided. It was easier because they would “normally have to go outside to smoke” (P021S) and take “fifteen minutes or so to roll a cigarette” (P003S). Also vaping allows people who use vapes the “option to take breaks in between whereas with a cigarette, you have to smoke the whole [cigarette]” (P025S). Many liked the “convenience” (P021S) of vaping. However, some found the device less easy and thought it may have malfunctioned. A few participants who previously smoked and subsequently vaped felt like it was like “transitioning to vaping all over again” (P007V) and found it harder to use compared to their usual devices which resulted in a negative experience.

When asked about the effectiveness of the device, some participants who smoked liked “the fact that you can adjust it if you want to” (P025S). This was more important for their experience than the flavour for some – P023S found “the voltage adjustment system made [vaping]… made it better […] turning on the voltage to make it less burny and horrible is a big upgrade, but the liquid isn’t particularly different from anything I’ve tried before.”

For both participants who smoked and participants who vaped “the cost benefits” (P020V) also make vaping appealing as “it costs a lot less money [than smoking]” (P024S). Another appeal of vaping is that it is seen as more socially acceptable than smoking. P017V began vaping because “it was nicer, and [you can] not be around your mates and stink of it, or whatever and it’s […] just a bit more socially acceptable.” P014V also said “it’s easier [to vape] when you’re around other people […] like sat outside in a pub area, something like that”. They also spoke of their dislike of being near others smoking because “that smoke’s wafting, wafting over onto other people, whilst with vaping that doesn’t really happen.”

Other reasons discussed for people who do not smoke and young people using e-cigarettes were “to get breaks at work” (P019V) and due to the behavioural aspects. P020V revealed that a friend of theirs “never smoked and all of a sudden now he’s just vaping, just because I think he likes the act of it”. P024S also admitted that they were 13 years old when they started smoking (in a country where flavoured tobacco was legal) and they “didn’t go for the flavoured products, I just used the regular ones because my friends were doing it and that kind of stuff, yeah.”

In a nationally representative survey, 11% of UK adults stated ease of use as a reason for using the e-cigarette device they used (Action on Smoking and Health (ASH), 2021) (Perski, Beard, & Brown, 2020). Patel and colleagues (2016) found 34% of US adults who vape cited e-cigarettes costing less than other tobacco products as a reason for using e-cigarettes. Using tank devices is also associated with higher risk of relapse compared with use of more effective modular devices (Brose, Bowen, McNeill, & Partos, 2019).

**References**

Abrams, D. B., Niaura, R., Brown, R. A., Emmons, K. M., Goldstein, M. G., & Monti, P. M. (2003). *The Tobacco Treatment Handbook: A Guide to Best Practices.* New York: Guildford Press.

Action on Smoking and Health (ASH). (2021). *Use of e-cigarettes (vapes) among adults in Great Britain.* . Retrieved from [https://ash.org.uk/wp-content/uploads/2021/06/Use-of-e-cigarettes-vapes-among-adults-in-Great-Britain-2021.pdf](about:blank)

Brose, L. S., Bowen, J., McNeill, A., & Partos, T. R. (2019). Associations between vaping and relapse to smoking: preliminary findings from a longitudinal survey in the UK. *Harm Reduction Journal, 16*(1), 76. doi:10.1186/s12954-019-0344-0

Patel, D., Davis, K. C., Cox, S., Bradfield, B., King, B. A., Shafer, P., . . . Bunnell, R. (2016). Reasons for current E-cigarette use among U.S. adults. *Preventive Medicine, 93*, 14-20. doi:[https://doi.org/10.1016/j.ypmed.2016.09.011](about:blank)

Perski, O., Beard, E., & Brown, J. (2020). Association between changes in harm perceptions and e-cigarette use among current tobacco smokers in England: a time series analysis. *BMC Medicine, 18*(1), 98. doi:10.1186/s12916-020-01565-2

Smets, J., Baeyens, F., Chaumont, M., Adriaens, K., & Van Gucht, D. (2019). When Less is More: Vaping Low-Nicotine vs. High-Nicotine E-Liquid is Compensated by Increased Wattage and Higher Liquid Consumption. *International Journal of Environmental Research and Public Health, 16*(5). doi:10.3390/ijerph16050723
