# Supplementary material for: Exploring the opinions and potential impact of unflavoured e-liquid on smoking cessation among people who smoke and smoking relapse among people who previously smoked and now use e-cigarettes: findings from a UK-based mixed methods study
Source: Harm Reduct J. 2024 May 3;21:90. doi: 10.1186/s12954-024-01003-z (PMC11067290; doi:10.1186/s12954-024-01003-z)
Supplement: Supplementary file 2 — Additional file 2. contains Supplementary Tables S1–S5. [file 12954_2024_1003_MOESM2_ESM.docx]

**Additional File 2: Supplementary Tables for “Exploring the opinions and potential impact of unflavoured e-liquid on smoking cessation among people who smoke and smoking relapse among people who previously smoked and now use e-cigarettes: Findings from a UK-based mixed methods study”**

**Supplementary Table 1. Qualtrics survey questions for participants who smoked**

| **Question** | **Response options** |
| --- | --- |
| How old are you (in years)? | Free text |
| Which of the following most accurately describes your gender? | Female  Male  Transgender  Intersex  Non-Binary  Prefer not to say  I would prefer to describe my gender in my own words [free text] |
| Which of the following most accurately describes your ethnicity? | White  Mixed / Multiple ethnicity  Asian / Asian British  Black/African/Caribbean/Black British  Other ethnicity [free text] |
| How many months or years have you smoked every day? | Less than 3 months  3-6 months  6-12 months  1-2 years  2-5 years  5+ years |
| How many cigarettes do you smoke a day? | Free text |
| Are you currently attempting to quit smoking? | No  Yes |
| Do you want to quit smoking? | Yes  No  Maybe |
| Below are some thoughts that smokers have about quitting. Please select one response that shows what you think about quitting. Please read each sentence carefully before deciding. | I have quit smoking.  I have quit smoking, but I still worry about slipping back, so I need to keep working on living smoke free.  I still smoke, but I have begun to change, like cutting back on the number of cigarettes I smoke. I am ready to set a quit date.  I definitely plan to quit smoking in the next 30 days.  I definitely plan to quit smoking in the next 6 months.  I often think about quitting smoking, but I have no plans to quit.  I sometimes think about quitting smoking, but I have no plans to quit.  I rarely think about quitting smoking, and I have no plans to quit.  I never think about quitting smoking, and I have no plans to quit.  I enjoy smoking and I have decided not to quit smoking for my lifetime. I have no interest in quitting. |
| Would you be willing to use an e-cigarette to quit smoking? | Yes  No |
| Are you planning to use an e-cigarette to quit smoking in the future? | Yes  No  Maybe |
| If flavoured e-liquids were removed from the market, and only tobacco, menthol/mint and unflavoured e-liquids were available, would you be willing to use an e-cigarette to quit smoking? | Yes  No  Maybe |
| Would you be willing to use an e-cigarette with an unflavoured e-liquid to quit smoking? | Yes  No  Maybe |
| Are you planning to use an e-cigarette with an unflavoured e-liquid to quit smoking? | Yes  No  Maybe |
| If flavoured e-liquids were removed from the market, and only tobacco, menthol/mint and unflavoured e-liquids were available, would you be willing to use an e-cigarette to quit smoking? | Yes  No  Maybe |
| Have you ever vaped (either tried or for prolonged use) in the past? | Yes  No |

**Supplementary Table 2. Qualtrics survey questions for participants who previously smoked and subsequently vaped**

| **Question** | **Response options** |
| --- | --- |
| How old are you (in years)? | Free text |
| Which of the following most accurately describes your gender? | Female  Male  Transgender  Intersex  Non-Binary  Prefer not to say  I would prefer to describe my gender in my own words [free text] |
| Which of the following most accurately describes your ethnicity? | White  Mixed / Multiple ethnicity  Asian / Asian British  Black/African/Caribbean/Black British  Other ethnicity [free text] |
| For how many months or years have you used an e-cigarette every day? | Less than 3 months  3-6 months  6-12 months  1-2 years  2-5 years  5+ years |
| How many times per day do you usually use your electronic cigarette? (Assume one “time” consists of around 15 puffs, or lasts around 10 minutes.) [enter number] | Free text |
| What strength nicotine do you use in your e-cigarette? | 0mg (Nicotine Free)  Between 1mg/ml and 3mg/ml (0.1%-0.3%)  Between 4mg/ml – 6mg/ml (0.4%-0.6%)  Between 7mg/ml – 9mg/ml (0.7%-0.9%)  Between 10mg/ml – 12mg/ml (1%-1.2%)  Between 13mg/ml – 15mg/ml (1.3%-1.5%)  Between 16ml/ml – 18mg/ml (1.6%-1.8%)  19mg/ml or more (1.9% or more) |
| How long ago did you quit smoking cigarettes? | Within the past three months  Between 3-6 months ago  Between 6-12 months ago  Over 12 months ago |
| For how many months or years did you smoke every day? | Less than 3 months  3-6 months  6-12 months  1-2 years  2-5 years  5+ years |
| When you were smoking daily, how many cigarettes did you smoke per day? [enter number] | Free text |
| What flavoured e-liquid(s) do you currently use? [free text box to include all] | Free text |
| Would you consider using an unflavoured e-liquid instead of your current e-liquid in the future (i.e., after this study)? | Yes  No |
| Do you plan to use an unflavoured e-liquid instead of your current e-liquid in the future (i.e., after this study)? | Yes  No |
| If flavoured e-liquids were removed from the market (e.g., no sweet or fruit flavours), and only tobacco, menthol/mint and unflavoured e-liquids were available, would you (tick all that apply): | Use unflavoured e-liquids  Use tobacco flavoured e-liquids  Use menthol/mint flavoured e-liquids  Stop using an e-cigarette  Start smoking cigarettes again  Make your own flavoured e-liquid  Buy flavoured e-liquid from an illegal store  Do not know  Other [free text] |
| If you had used an unflavoured e-liquid when you quit smoking, do you think you would have successfully quit? | Yes  No  Maybe |

**Supplementary Table 3. Interview schedule / topic guide for participants who smoked**

| **Questions** | **Prompts** |
| --- | --- |
| Have you tried quitting smoking before? | - Why/why not? - Why did it not work? |
| Have you tried vaping before? | - Why/why not? - How much and how often did you vape? - How long did you vape for? - Why did you stop? |
| How motivated to quit smoking do you feel? | - Why/why not? |
| After trying vaping using the e-liquid that we provided, what were your first impressions of vaping? | - To what extent did you enjoy vaping with the e-liquid we provided? - To what extent did it satisfy your cravings for cigarettes? - Was there anything you liked or disliked about the e-liquid we gave you? |
| What did you think about the taste and flavour of the e-liquid? | - How would you describe the flavour? - Would you describe it as unflavoured/flavourless? - How much did you like/dislike the taste? |
| How does using the e-cigarette and the e-liquid provided compare to smoking? | - Does it provide the same feeling/result? Why/why not? - Does it satisfy your urge to smoke/ smoking cravings? Why/why not? |
| Has your opinion about vaping and unflavoured e-liquids changed after trying the e-liquid? | - Why do you think your opinion has changed? - What did you think before, and what do you think now? |
| After using the e-cigarette we have provided, would you be willing to use an e-cigarette to quit smoking? | - Why/why not? |
| After using the e-liquid we have provided, would you be willing to use an e-cigarette with an unflavoured e-liquid to quit smoking? | - Why/why not? |
| How likely would you be to continue vaping after this study using other e-liquid flavours such as candyfloss, bubblegum, apple? | - Why/why not? |
| If flavoured e-liquids were removed from the market, and only unflavoured, tobacco or menthol/mint flavours were available would it affect how likely you were to use an e-cigarette in a quit attempt? | - Would you use an e-cigarette when trying to quit smoking in this scenario? - What flavour e-liquids would you choose to vape and why? |
| If flavoured e-liquids were removed from the market, and only unflavoured, tobacco or menthol/mint flavours were available, how likely is it that you would continue to smoke? | - Would you continue smoking in this scenario? - Why do you think you [would/wouldn’t] start smoking? |
| Some countries have removed or are wanting to remove flavoured e-liquids from the market as they think it can encourage smoking in young people – to what extent do you think this would be a good idea? | - Why? - Should we ban flavours in the UK? |
| Are there any other comments you’d like to add? |  |

**Supplementary Table 4. Interview schedule / topic guide for participants who previously smoked and subsequently vaped**

| **Questions** | **Prompts** |
| --- | --- |
| Did the variety of e-liquid flavours available affect your decision to quit smoking or use an e-cigarette to quit? | - Why/How did it affect your decision? - To what extent did you find the variety of flavours available appealing when you decided to start vaping? - How much do you think this influenced your decision to start vaping? |
| Do you think your decision to switch or maintain smoking abstinence/e-cig use would have been affected if only unflavoured, tobacco, or menthol flavours had been available? | - Why/why not? - How much do you think this influenced your decision to continue vaping? - How much do you think this influenced your decision to stay quit/not smoke? |
| What did you think about the e-liquid that we provided? | - To what extent did you enjoy using the e-liquid? - Was there anything you liked or disliked about the e-liquid we gave you? - Was it better or worse than you expected and why? - How does it compare to smoking? |
| What did you think about the taste and flavour of the e-liquid? | - How would you describe the flavour? - Would you describe it as unflavoured/flavourless? - How much did you like/dislike the taste? |
| How did the e-liquid compare to your normal e-liquid? | - Is it better or worse? - What is it that you prefer about the e-liquid you prefer? |
| How likely is it that you would use unflavoured e-liquid again? | - Why? |
| Has your opinion about unflavoured e-liquids changed after trying the e-liquid? | - Why do you think your opinion has changed? - What did you think before, and what do you think now? |
| Would you consider using unflavoured e-liquid instead of your current e-liquid in the future? | - Why/Why not? - Do you plan to use an unflavoured e-liquid instead of your current e-liquid in the future? - Why/why not? |
| If flavoured e-liquids were removed from the market, and only unflavoured, tobacco or menthol/mint flavours were available would it affect your vaping behaviour in any way? | - Would you continue to vape? - What flavour e-liquids would you choose to vape and why? - Would you get your e-liquids from the same source as you do now? - Where/how would you get your e-liquids? |
| If flavoured e-liquids were removed from the market, and only unflavoured, tobacco or menthol/mint flavours were available, how likely is it that you would start smoking again? | - Why do you think you [would/wouldn’t] start smoking? |
| How much do you think the removal of flavoured e-liquids from the market would affect how often you vape? | - Why |
| If you had used an unflavoured e-liquid when you quit smoking, do you think you would have successfully quit? | - Why |
| Some countries have introduced or are wanting to remove flavoured e-liquids from the market as they think it can encourage vaping in non-smokers and youth, which may also lead to smoking – To what extent do you think this would be a good idea? | - Why? - Should we ban flavours in the UK? |
| Are there any other comments you’d like to add? |  |

**Supplementary Table 5. Self-reported flavours currently used by each participant who vapes.**

| **Participant** | **Flavours currently used** |
| --- | --- |
| P004V | Strawberry, Lemon, Raspberry, Blueberry |
| P006V | Blackcurrant, Lime, Cola |
| P007V | Various: Fruit Flavours, Pastry flavours |
| P009V | Various Berry Flavours |
| P013V | Fruit |
| P015V | Fruity |
| P014V | Berry |
| P018V | Fruit Flavours |
| P017V | Fruit Flavoured, Mango, Pineapple |
| P016V | Fruit, Raspberry |
| P019V | Strawberry, Tropical, Mango |
| P020V | Strawberry |
